# Safety and efficacy of reduced dosage ketoprofen with or without tramadol for long-term treatment of osteoarthritis in dogs: a randomized clinical trial

**DOI:** 10.1186/s12917-019-1960-3

**Published:** 2019-06-25

**Authors:** Beatriz P. Monteiro, Cedric Lambert, Elena Bianchi, Jean Pierre Genevois, Giulio Soldani, Eric Troncy

**Affiliations:** 1 Department of Biomedical Sciences, GREPAQ (Groupe de recherche en pharmacologie animale du Québec), Faculty of Veterinary Medicine – Université de Montréal, 3200 Sicotte Street, Saint-Hyacinthe, QC J2S 2M2 Canada; 2 Department of Companion Animals, Vet-Agro Sup, Lyon, France; 3 Department of Pharmacology, College of Veterinary Medicine, University of Pisa, Pisa, Italy

**Keywords:** Adverse-effects, Chronic pain, Dog, Ketoprofen, Non-steroidal anti-inflammatory drug, Tramadol, Non-conventional analgesic, Efficacy, Safety

## Abstract

**Background:**

This study aimed to evaluate the safety and efficacy of reduced-dosage ketoprofen with or without tramadol in dogs. Five healthy dogs receiving standard-dosage ketoprofen (2 mg/kg SC, then 1 mg/kg PO daily) comprised Group A. Twenty dogs with osteoarthritis were randomized to receive reduced-dosage ketoprofen (0.5 mg/kg SC once; 0.25 mg/kg PO daily) alone (Group B) or in combination with tramadol (5 mg/kg/day PO) (Group C). Treatments were administered for 28 days. Platelet aggregation time (PAT), gastrointestinal (GI) endoscopy and glomerular filtration rate (GFR) were performed up to 60 days after treatment initiation. Pain was scored using a validated clinical metrology instrument up to D120. Data were analyzed with general linear mixed model for repeated measures (α = 0.05).

**Results:**

PAT was not different between groups but was increased with time for all groups. GI lesion scores were higher in Group A than Groups B and C (day 28; *P* = 0.005) and were increased with time for Group A (*P* = 0.005). GFR was lower in Group A than Groups B and C (day 28; *P* < 0.01) and were decreased with time for group A (*P* < 0.001). Standard-dosage ketoprofen administration resulted in clinically relevant adverse effects. Pain score decreased in both treated groups (B and C) from D0 to D28. Need of rescue analgesia from D29 to D120 was higher in Group B than in Group C (*P* = 0.039).

**Conclusions:**

The long-term safety profile of reduced-dosage ketoprofen is similar whether the drug is administered alone or in combination with tramadol to dogs with osteoarthritis. Analgesic efficacy of the combination looks attractive.

**Electronic supplementary material:**

The online version of this article (10.1186/s12917-019-1960-3) contains supplementary material, which is available to authorized users.

## Background

Osteoarthritis (OA) is a progressive degenerative disease of synovial joints characterized by structural and functional changes secondary to inflammatory, biomechanical and metabolic components [1, 2]. Except for the cartilage, all joint’ structures are innervated, and pain is a major clinical feature of OA [3]. In affected patients, nociceptors of joint afferents are chronically exposed to inflammatory mediators resulting in peripheral sensitization. The constant nociceptive input from the periphery to the spinal cord further contributes to central sensitization [4].

Currently, there are no disease-modifying therapies with strong evidence of efficacy in canine OA; therefore, its management is based on relieving symptoms and improving function [5, 6]. Non-steroidal anti-inflammatory drugs (NSAIDs) produce analgesic, anti-inflammatory and antipyretic effects primarily by the inhibition of the expression of cyclooxygenase (COX) enzymes in cell membranes that are responsible for the synthesis of inflammatory mediators. Orally administered NSAIDs and related prostaglandin E_2_ receptor 4 (EP_4_) antagonists, such as grapiprant, remain the first-line pharmacological therapy in OA [5]; however, the use of NSAIDs is associated with a risk of adverse events, notably gastrointestinal (GI), hepatic, hemostatic and renal [7]. Ketoprofen is a non-specific COX inhibitor. Despite early concerns related to potential hemostatic, renal and GI toxicity after the preoperative [8, 9] or long-term [10] administration of the standard dosage of ketoprofen, a superior safety profile was observed when this drug was administered at a reduced dosage (0.25 mg/kg) for long-term use in dogs [11, 12]. Research has shown that these adverse events are more likely to occur with higher NSAID dosing and in individuals with a pre-existing risk for complications [13]. Therefore, NSAID dose reduction appears logical for dogs [14], and for cats [15] affected by chronic painful conditions.

Tramadol is a centrally-acting analgesic with a complex pharmacological profile. The main mechanisms of action of tramadol include agonism of μ-opioid receptors and inhibition of norepinephrine and serotonin reuptake. While more active, these central monoamine neuromediators accentuate endogenous inhibitory pain control. Other mechanisms include inhibition of α_2_-adrenoceptors, neurokinin 1, muscarinic, nicotinic acetylcholine and N-methyl-D-aspartate receptors [16]. This drug is widely used for the treatment of OA-related pain in humans due to its positive effects on physical function and tolerability [17]. The efficacy and safety profile of tramadol after long-term treatment in dogs have been rarely investigated and recent evidence suggests a lack of efficacy on signs of pain (subjective questionnaire) and orthopedic dysfunction (kinetic assessment), when administered alone in dogs with OA [18]. A previous report mentioned an improvement on the same subjective pain questionnaire, but not on kinetic gait analysis for dogs with hip OA receiving tramadol [19].

Tramadol is overall well tolerated in dogs [20]. Adverse effects of tramadol overdose include restlessness, difficulty walking, salivation, vomiting, tremors, and convulsions. Administration of 40 mg/kg per day to 8 dogs was well tolerated for 1 year, with mydriasis and reduced body weight observed [21]. Adverse effects such as nausea and anorexia, and occasionally sedation, have been reported in dogs with routine dosages of tramadol [20]. In OA, inflammatory and nociplastic (centralized) pain processes play a critical role in the clinical manifestation of the disease. Thus, a mechanistic rationale for pharmacotherapies with the concomitant administration of NSAID and centrally-acting analgesic such as tramadol exists. Nevertheless, a concern of increased risk of GI adverse events due to this co-administration also exists and has been highlighted in reports both in humans [22, 23], dogs [24] and cats [25]. The rationale behind this interaction is that added to the GI effects of NSAIDs, serotonin is known to modulate gastric acid secretion and platelet aggregation. The former would contribute to gastric mucosal lesions, and the latter would impact mucosal healing [20, 23].

In dogs, a study investigating the effects of the co-administration of tramadol and indomethacin in an ex-vivo model did not detect any deleterious effects on the gastric mucosa [26]. Similarly, dogs treated with meloxicam, a COX-2 preferential NSAID, or tramadol (4–5 mg/kg q12h PO), or the combination of both for 10 days revealed no differences in GI safety between treatments by means of gastroscopy [27]. Yet, the safety profile of the combination of reduced-dosage ketoprofen and tramadol in dogs with naturally-occurring OA remains unknown. We opted for a non-specific COX inhibitor as this type of NSAID is supposed to be potentially more deleterious. If the ketoprofen – tramadol association were to be safe, it would be reasonable to extrapolate that any NSAID – tramadol association is also likely to be safe.

The authors hypothesized that the administration of a reduced-dosage ketoprofen as single agent would have a superior safety profile when compared with the standard dosage ketoprofen or with the co-administration of reduced-dosage ketoprofen and tramadol. The goal of this study was to evaluate the safety and efficacy profile of a reduced-dosage ketoprofen with or without tramadol in client-owned dogs with OA by comparison with healthy dogs receiving the standard dose. Outcome measures included physical and laboratory examinations, coagulation profile, GI endoscopy and glomerular filtration rate (GFR) measurement for safety, and a validated subjective clinical metrology instrument for efficacy. For ethical reason, the standard dosage ketoprofen could be tested with daily administration over 28 days, only on laboratory (continuously monitored) dogs to establish gold standard of ketoprofen toxicity. It was hypothesized that the reduced-dosage ketoprofen would induce lower side effects than this gold standard, allowing to compare between these two extremes the effect of a tramadol and reduced-dosage ketoprofen combination. Efficacy was tested only on client-owned dogs with OA in a randomized prospective controlled double-blinded clinical trial.

## Results

Twenty-five dogs aged from 2 to 12 years old and weighing from 10 to 56 kg were included in the study (Table 1). All dogs included in groups B and C presented radiographic and clinical signs of OA, without any statistical difference between groups. Affected joints included bilateral hips (*n* = 6), right stifle (*n* = 5), bilateral hips and shoulders (*n* = 4), bilateral hips and right or left elbow (*n* = 3), bilateral elbows (*n* = 1) and right shoulder (*n* = 1). Clinical signs as reported by the owners started between 1 and 12 months prior to inclusion in the study. The following breeds were represented: Labrador Retriever (*n* = 7), German Shepherd (*n* = 7), Rottweiler (*n* = 3), Boxer (*n* = 1), Fawn Brittany (*n* = 1) and Fox Terrier (*n* = 1). At D0, there were no statistical differences among the 3 groups with regards to gender, age, standard laboratory analyses (complete blood count (CBC) including white blood cells differential count – *data not shown* –, renal and hepatic profile), and platelet aggregation, GI endoscopic score and GFR (Table 1).

**Table 1:** Demographic data, laboratory analyses, gastrointestinal endoscopic scores and glomerular filtration rate

	Reference range	Group A	Group B	Group C
Sex				
Male (n)		2	7	6
Female (n)		3	3	4
Age (years)		4.4(0.5)	10.3(1.8)	9.1(3.7)
Body weight (kg)		12.2(1.9)^a^	36.4(7.4)	38.6(11.8)
Osteoarthritis radiographic score (no unit)	0–9	Not applicable	6.7(3.3)	5.8(2.9)
Affected joints				
• Bilateral hips			*n* = 3	*n* = 3
• Stifle (right)			*n* = 2	*n* = 3
• Bilateral hips and shoulders			*n* = 1	*n* = 3
• Bilateral hips and elbow			*n* = 2	*n* = 1
• Bilateral elbows			*n* = 1	
• Shoulder (right)			*n* = 1	
Duration of clinical signs evolution (months)	As reported by the owners		4.1(1.8)	5.6(2.4)
Red blood cell count (M/mm^3^)	5.5–8.5	6.8(0.7)	7.2(1.1)	7.5(0.6)
Hematocrit (%)	37–54	50.9(4.6)	44.6(7.8)	49.4(6.4)
Hemoglobin concentration (g/dL)	12–18	15.9(1.3)	16.4(2.9)	15.8(1.9)
Mean corpuscular volume (μ^3^)	60–77	75.2(0.8)	67.6(1.0)	71.2(3.4)
Mean corpuscular hemoglobin concentration (g/dL)	31–36	31.3(0.2)	34.3(1.8)	32.6(3.1)
Leucocyte count (m/mm^3^)	6–17	5.5(0.6)	7.2(3.2)	9.8(2.6)
Platelet count (m/mm^3^)	160–525	450.8(95.2)	448(122.2)	384.8(118.4)
Blood urea nitrogen (mEq/L)	2.1–9.7	4.2(1.1)	5.8(1.4)	4.8(0.9)
Creatinine (μmol/L)	42–135	62(10.8)	93.3(14.4)	82.5(13.2)
Alkaline phosphatase (UI/L)	0–200	61.0(15.1)	53.6(28.8)	92.4(86.2)
Alanine aminotransferase (UI/L)	0–130	52.2(38.4)	46.2(28.8)	38.2(16.7)
Platelet aggregation time (seconds)	0–213	133.6(17.7)	178.2(32.4)	168.7(38.5)
Gastrointestinal endoscopic score (no unit)	0–50	2.4(1.8)	1.6(1.5)	1.1(1.6)
Glomerular filtration rate (mL/min/m^2^)	55–117.1	97.2(7.15)	87.9(14.1)	94.6(12.9)

Data from baseline evaluation (D0). Group A refers to healthy dogs and Groups B and C refer to dogs with osteoarthritis. Data are presented as mean(SD)

^a^Difference statistically significant among groups

### Standard bioanalyses

Red blood cell count was not different between groups at any time-point. However, all three groups presented a significant decrease in red blood cell count (*P* < 0.03) and hemoglobin concentration (*P* < 0.04) at D7 and D28 when compared with D0. A limited number of dogs in groups B (*n* = 3) and C (*n* = 2) revealed values inferior to the lower limit (5.5 M/mm^3^) of red blood cell count at D7 and D28, while this was observed in all dogs in group A (*n* = 5). Group A presented a within-time difference with increased hematocrit (*P* = 0.04) and mean corpuscular volume (*P* = 0.008) and decreased mean corpuscular hemoglobin concentration (*P* = 0.025) at D28 when compared with D7. No statistically significant change was noted in groups B and C. Leucocyte count and differential, as well as platelet count did not show any statistical difference within-time or between-groups.

There were no differences within-time or between-groups for blood urea nitrogen, creatinine, and both hepatic enzymes alkaline phosphatase and alanine aminotransferase.

### Coagulation profile

**Fig. 1:**
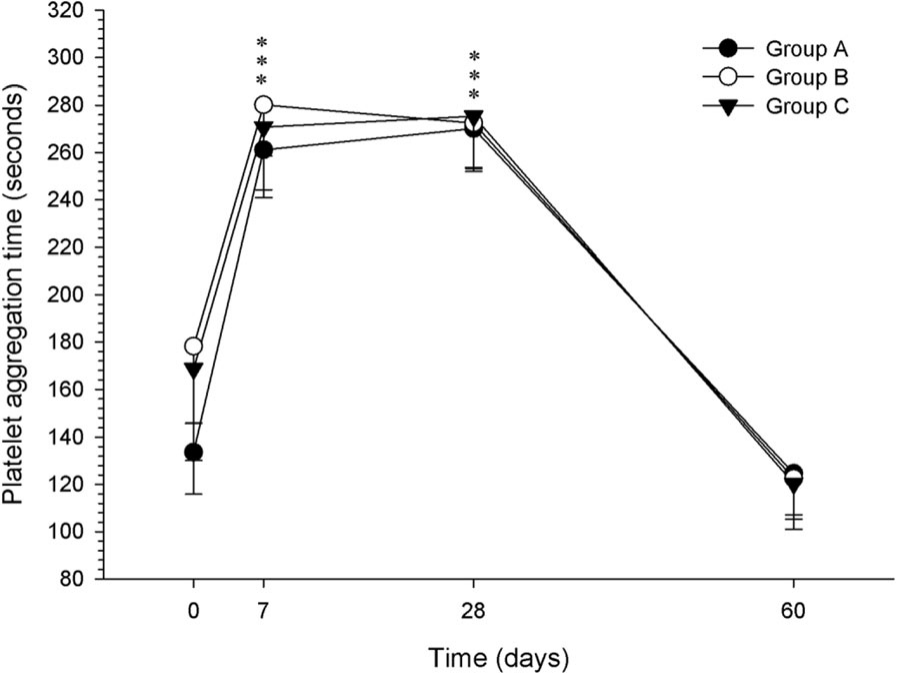
Platelet aggregation time (in seconds) of healthy dogs (group A) and those with osteoarthritis (groups B and C). Evaluations were performed at baseline (D0) and after 7, 28 and 60 days of treatment over 28 days with standard dosage ketoprofen (Group A, *n* = 5), or reduced dosage ketoprofen alone (Group B, *n* = 10) or with tramadol (Group C, n = 10). Data are presented as mean. Error bars represent standard deviation. *Indicates significant within-time difference when compared with baseline (day 0) and day 60

Platelet aggregation time (PAT) was not different among groups at any time. Within-time differences were observed for groups A, B and C (*P* = 0.01, *P* = 0.001 and *P* = 0.005, respectively), with a significant increase at D7 and D28, compared to D0 and D60 (Fig. 1). Buccal mucosal bleeding time was increased in most dogs but did not reach abnormal values for any dog.

### Gastrointestinal endoscopy

**Fig. 2:**
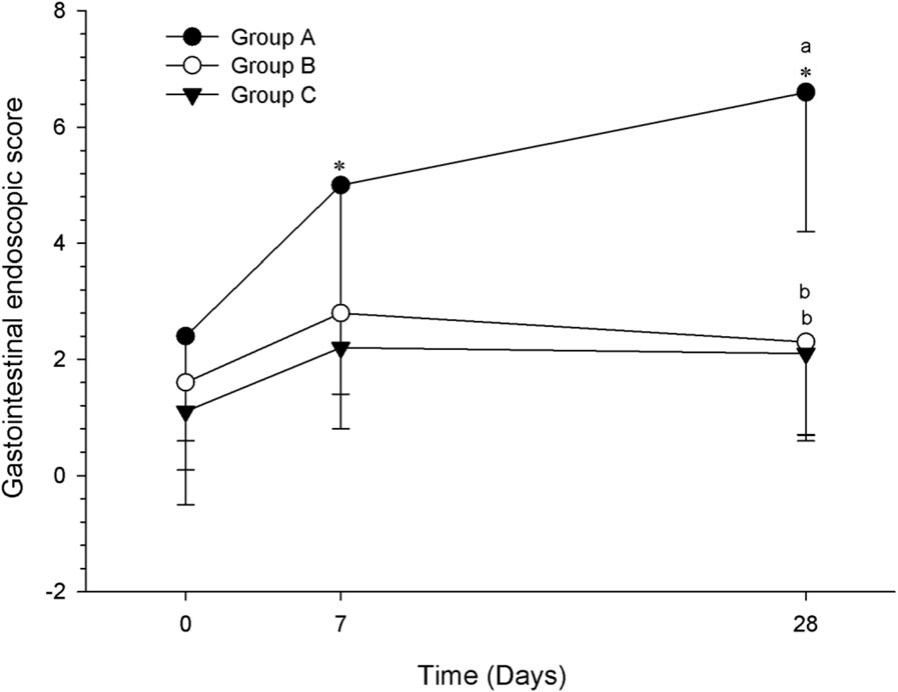
Gastrointestinal endoscopic score of healthy dogs (group A) and those with osteoarthritis (groups B and C). Evaluations were performed at baseline (D0) and after 7 and 28 days of treatment with standard dosage ketoprofen (Group A, *n* = 5), or reduced dosage ketoprofen alone (Group B, *n* = 10) or with tramadol (Group C, *n* = 10). Data are presented as mean. Error bars represent standard deviation. *Indicates significant within-time difference when compared with baseline (day 0). Different letters indicate statistically significant differences between groups

Only group A presented a statistically significant within-time increase in GI endoscopic score (*P* = 0.005). The pairwise comparison for group A demonstrated that scores from D0 were different from D7 and D28 (*P* < 0.025) (Fig. 2). Moreover, at D28, group A was statistically different from both groups B and C (*P* = 0.005). The severity of lesions was different among groups. In groups B and C, at D7 and D28, only petechiae were observed, whereas in group A, suffusion was observed in 1/5 dog on D7 and D28, and erosions and non-perforating ulcer were observed in 1/5 dog on D28. In addition, > 20 petechiae were seen at D7 and D28, respectively, in 4/5 and 3/5 dogs in group A, 2/10 and 1/10 dogs in group B, and 2/10 and 2/10 dogs in group C. None of the dogs developed a perforating ulcer. Most lesions were observed in the pyloric antrum and proximal duodenum, and rarely at the body of the stomach. No lesions were observed in the esophagus.

### Glomerular filtration rate

**Fig. 3:**
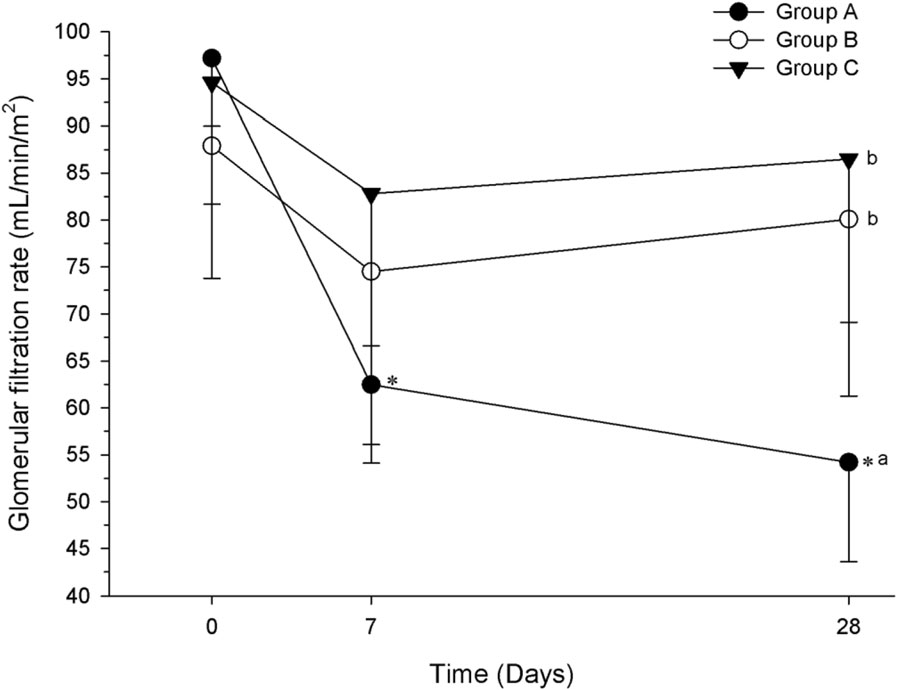
Glomerular filtration rate (mL/min/m^2^) of healthy dogs (group A) and those with osteoarthritis (groups B and C). Evaluations were performed at baseline (D0) and after 7 and 28 days of treatment with standard dosage ketoprofen (Group A, *n* = 5), or reduced dosage ketoprofen alone (Group B, n = 10) or with tramadol (Group C, *n* = 10). Data are presented as mean. Error bars represent standard deviation. *Indicates significant within-time difference when compared with baseline (day 0). Different letters indicate statistically significant differences between groups

There was a significant decrease within-time in GFR for group A (*P =* 0.01) at D7 and D28 when compared with D0 (*P* < 0.001), (Fig. 3). The between-groups comparison was significant at D28, with lower GFR in group A compared with groups B and C (*P* < 0.01).

### Outpatient monitoring of adverse events

Sixteen out of 20 owners from groups B and C returned the completed questionnaire at the end of the study. From these, eight dogs developed adverse events; Group B: vomiting in different occasions at D5, D27 (*n* = 1); one episode of diarrhea at D4 (*n* = 1) and D6 (*n* = 1); Group C: flatulence at D2 (*n* = 1), repeated flatulence at D4, D8, D13, D19, D23 (*n* = 1); one episode of diarrhea at D8 (*n* = 1); cough of 24 h duration at D20 (*n* = 1); constipation of 48 h duration at D28 (*n* = 1). The dog from group C that developed diarrhea was withdrawn from the study for 48 h for which period, ketoprofen and tramadol were stopped, and supportive treatment was administered (sucralfate). The dog recovered during this period and was reincluded in the study without any recurrence of clinical signs.

### Pain scoring

Table 2 is presenting the evolution of pain scoring established by the clinical metrology instrument assessment in OA dogs (Groups B and C). At baseline, there was no difference between Groups B and C for the pain score. From D0 to D28, both groups presented a parallel evolution with no difference between groups, but a significantly decreased pain score in both groups at D28 (compared to D0). From D29 to D120, the within-time evolution in Group B was really stable, where the Group C pain score tended to show a declining slope (without statistical difference). Whereas all criteria presented a parallel evolution with no difference between groups from D0 to D28, some of them, namely Lameness while Trotting, Reaction to the Mobilization of the affected limb (joint) and Intensity of this reaction, differed greatly between groups from D29 to D120, all three presenting a deterioration in the Group B. The between-groups difference in pain was objectified by the need for rescue analgesia: Whereas one dog from Group B did not require rescue analgesia from D29 to D120, at least 50% of Group C dogs did not present any OA crisis over the same period (Table 3). The difference in OA crisis occurrence was statistically significant (Chi-Square *P*-value = 0.039).

**Table 2:** Evolution of pain score established by the clinical metrology instrument, Standardized Veterinarian Arthritis Pain Scale (SVAPS, assessed by a blinded (to treatment) veterinarian in both treated groups of osteoarthritic dogs

Time-points	D0	D7	D28	D60	D120
Group B	17.2(8.4)^a^	11.8(6.2)^a,b^	6.4(4.1)^b^	6.8(3.8)^b^	6.6(3.2)^b^
Group C	14.8(7.9)^a^	9.2(6.4)^a,b^	4.8(3.2)^b^	3.9(2.8)^b^	2.9(2.8)^b^

Different letters in superscript mean different time-point values in the same group. Group B refers to dogs with osteoarthritis treated from D0 to D28 with ketoprofen alone. Group C refers to dogs with osteoarthritis treated from D0 to D28 with the ketoprofen – tramadol association. Data are presented as mean(SD)

**Table 3:** Number of osteoarthritis pain crisis occurring over the D29 – D120 period for both treated groups

	No OA crisis	Dogs with at least one OA crisis
Group B	1	7
Group C	5	3

Group B refers to dogs with osteoarthritis treated from D0 to D28 with ketoprofen alone. Group C refers to dogs with osteoarthritis treated from D0 to D28 with the ketoprofen – tramadol association

## Discussion

This study evaluated the safety of administration of reduced dosage ketoprofen alone (Group B) or in combination with tramadol (Group C) by comparison with standard dosage ketoprofen (Group A), as well as the efficacy of treatment in both osteoarthritic groups (B and C). Results showed an absence of clinically important adverse effects in groups B and C with regards to GI and renal function, whereas in group A, marked GI and renal effects were observed. Hemostatic function was decreased for all three treatment groups in this study.

Routine laboratory analysis such as CBC and serum chemistry profile did not reveal clinically relevant changes in time or between groups after drug administration with a few exceptions. The decrease in red blood cell count and hemoglobin concentration at D7 and D28 for all groups and the anemia observed in some dogs from groups B (30%) and C (20%) and in all dogs from group A (100%) likely reflect GI bleeding which was more severe in group A. Group A also revealed additional changes consistent with a macrocytic regenerative anemia in response to the presumed GI bleeding. These findings contrast with other studies evaluating the safety of standard [12] and reduced dosage [28] ketoprofen administered in dogs for 30 days where no changes in hematological or serum chemistry profile were recorded. On the other hand, the lack of changes in hepatic enzymes herein is not surprising in dogs with no evidence of pre-existing hepatic disease. Several studies failed to detect significant changes during or after long-term administration of ketoprofen [10] or other NSAIDs [29, 30] in dogs. It is now well accepted that hepatic adverse effects are more likely to be an idiosyncratic reaction to the drug rather than intrinsic hepatotoxicity [7, 31].

Analysis of primary hemostasis by use of platelet function analyzer (PFA) revealed increased PAT with values above reference range in all groups at D7 and D28. A difference between groups was not detected. These findings indicate that the effects of ketoprofen on PAT are neither dose-dependent nor affected by the combination with tramadol. Nevertheless, buccal mucosal bleeding time was normal in dogs presenting with increased PAT; thus, the clinical significance of these findings is questionable and indicates that buccal mucosal bleeding time is insensitive to the NSAID effect on PAT [32]. The literature is controversial with regards to hemostasis and NSAIDs in dogs and this might be explained by different drugs, dosage regimen, animal population, and primary or secondary hemostatic function tests (i.e., buccal mucosal bleeding time, PFA, thromboelastography, prothrombin time, activated partial thromboplastin time, etc.). For example, when 10 healthy dogs were administered aspirin, carprofen, deracoxib or meloxicam for 7 days, platelet function assessed by PFA did not differ before and after treatment [33]. Similarly, when reduced [28] or standard [34] dosage ketoprofen was administered for 30 days to healthy dogs, buccal mucosal bleeding time did not change and was always within reference range. Controversially, when etodolac, meloxicam, carprofen, ketoprofen or flunixin were administered for 90 days to healthy dogs, meloxicam, carprofen, ketoprofen and flunixin showed increased bleeding or clotting times at days 30, 60, and/or 90 when compared to baseline [10]. Finally, when ketoprofen was administered preoperatively to female dogs undergoing ovariohysterectomy, a significant decrease in platelet aggregation was observed, although buccal mucosal bleeding time did not change [9]. Inhibition of COX-1 and consequent synthesis of thromboxane A2 results in impaired platelet adhesion which is NSAID-dependent [7]. However, it seems from most studies that these effects are not clinically meaningful as bleeding time was rarely affected after NSAID administration.

Gastrointestinal endoscopy revealed increased lesion scores in group A at D7 and D28 when compared with baseline. Indeed, scores for group A at D28 were higher than groups B and C. These results suggest that GI lesions were ketoprofen dose-dependent and did not change when tramadol was added to the protocol. Similar findings were recorded when healthy dogs were administered placebo, meloxicam, tramadol or meloxicam-tramadol for 10 days [27]. In the latter study, no difference between groups was found in terms of GI endoscopy, fecal occult blood test and clinical monitoring. However, in the meloxicam-tramadol group, GI endoscopic scores were significantly increased at day 6 when compared with baseline values [27]. In the present study, the percentage of dogs from group A with GI lesions is similar to that observed in other studies using the standard dosage ketoprofen [34, 35]. The anatomical distribution of GI lesions was also similar to previous studies and the lack of esophageal lesions is not surprising since it does not depend on prostaglandins for mucosal protection [34, 35].

Glomerular filtration rate was decreased after treatment in dogs from group A which was different from Groups B and C at D28. These findings indicate that decreased GFR is ketoprofen dose-dependent and does not seem to be affected by the addition of tramadol. Similar findings are reported in the literature. In a previous study in which dogs undergoing castration were administered standard dosage ketoprofen, carprofen or placebo preoperatively, endogenous creatinine clearance was decreased 24 h after surgery in dogs receiving an NSAID when compared with placebo [8]. In that study, creatinine clearance was within normal limits and preoperative values were not available. In the study herein, despite decreases in GFR, values also remained within normal limits. Nevertheless, it seems clear that reduced dosage ketoprofen generates less renal dysfunction when compared with the standard dosage due to the lack of decrease in GFR in the former. Similarly, in another study in which dogs were administered reduced dosage ketoprofen or placebo for 30 days, no difference was found between groups nor within time in effective renal plasma flow and GFR [28]. These findings highlight the importance of GFR in the evaluation of renal function due to its higher sensitivity to assess renal function when compared with serum urea and creatinine since these only become increased when renal damage is already well advanced [30]. This is highlighted by the lack of changes on blood urea nitrogen and creatinine in dogs from group A in this study.

Few outwardly detectable adverse-effects were recorded for dogs from groups B and C; those that were reported were mild and predominantly related to the GI tract. Unfortunately, some adverse-effects from group A dogs could have been missed (twice daily clinical observations), although one might suspect that they would be more frequent and severe due to the GI endoscopic findings. In a previous study in which dogs were administered meloxicam in combination with tramadol for 10 days, no difference on the incidence of clinically monitored adverse events was found [27]. Finally, in dogs with cancer pain being administered a NSAID with metamizole, metamizole with tramadol or a NSAID with metamizole and tramadol, the incidence of adverse effects was greater in dogs receiving NSAID with metamizole than in other groups after 7 days of treatment [36]. Vomiting, drowsiness, diarrhea and constipation were the most commonly observed adverse effects in those dogs [36].

The effects of tramadol on gastric mucosa have been investigated in people and animal models. Tramadol has been shown to inhibit the secretion of gastric acid via inhibition of muscarinic type 3 receptors resulting in increased gastric pH after preoperative intramuscular [37] and intravenous administration [38], but not after oral administration [39] in people. In addition, serotonin is expected to be upregulated after tramadol treatment and is also known to affect gastric secretion [40]. Furthermore, the inhibition of serotonin reuptake potentially exerts an inhibitory effect on platelet activation [41]. All these factors can negatively affect gastric mucosa hemostasis, although they have not been clearly elucidated. For example, in dogs, an ex vivo study evaluated the effect of indomethacin, tramadol, or both on gastric barrier function, prostanoid production and COX expression, and found that there was no apparent interaction between both drugs suggesting that if there is an adverse interaction of the two drugs in vivo, it is unlikely to be via prostanoid inhibition [26]. Another questionable avenue is the GI effects of tramadol-derived metabolites, which are recognized to be species-specific.

Even though the underlying mechanisms of the synergism between NSAIDs and tramadol, particularly in the GI tract, are not well understood, the study herein and other studies [26, 27, 36] did find minor (if any) deleterious effects after the administration of this combination in dogs. Nevertheless, their clinical relevance must always be evaluated, as they could result in cessation of the treatment.

Additionally, one should take into account the questionable efficacy of tramadol alone in dogs diagnosed with radiographic OA [18]. Recent studies indicated that tramadol seems to produce analgesic effects in cats [42], but not (or slightly) in dogs [18, 19]. In feline OA, benefits of tramadol tested alone over 19 days [42] or in combination with meloxicam over 25 days [25] on laboratory cats emerged with improved kinetics and mobility (telemetered motor activity), and decreased nociplastic hypersensitivity, all objective sensory outcomes. This result was later translated on client-owned OA cats’ mobility and subjective questionnaire [43]. Differences in effect between both species is likely explained by a slower rate of formation of the active metabolite by the liver (3.9-fold) as well as shorter elimination half-life and lower concentrations of O-desmethyltramadol in dogs when compared with cats [44, 45]. In previous studies, it has to be noted the short duration of treatment with tramadol alone in dogs affected by advanced (radiographically present) OA, for 10 [18], and 14 days [19]. In the present study, the duration of treatment with a single low daily dose of slow-release tramadol [44] associated to an NSAID was really longer (28 days), and the subjective assessment with a validated clinical metrology instrument showed a clear efficacy, accentuated over time. Pain is defined as ‘an unpleasant sensory and emotional experience associated with actual or potential tissue damage, or described in terms of such damage’ [46]. It could be hypothesized that the assessment outcomes used in previous studies [18, 19] for testing tramadol alone efficacy were more reflective about the sensory component of pain, whereas tramadol with its specific metabolism in dogs could be more relevant to the pain affective component. This would be in agreement with a recent survey conducted in France through the *CAPdouleur* network (www.capdouleur.fr) where 160 practitioners responded to frequently use tramadol for canine OA (118 responses) and considered its analgesic efficacy as ‘satisfying or very satisfying’ for 86.7% of responders. Finally, the persistence of analgesic efficacy (no OA pain crisis with high limitation in mobility / activity) from D29 to D120 was present in at least 50% of dogs in Group C having received the ketoprofen – tramadol association. As all owners but one in Group B responded that their dog required rescue analgesia (*n* = 7) over the same period, this result is suggestive of a synergic effect in efficacy for the NSAID – tramadol combination.

One limitation of this study is the inclusion of research Beagle dogs for the standard dose of ketoprofen; thus, observers were not blinded to this treatment group. On the other hand, it would have not been possible to include client-owned dogs in this group due to the increased risk of adverse-effects as this approach would have been unethical. In addition, distribution of dogs in the three groups was homogenous, highlighted by the lack of differences among groups at baseline except for the body weight (BW). Differences in BW were related to group A being composed of Beagles (10 to 15 kg) which weighed less when compared with dogs in groups B and C. Another limitation is the lack of a group receiving tramadol only. It is possible that most of the adverse effects observed, notably from GI endoscopy, are primarily due to ketoprofen; however, it is impossible to confirm this without evaluating the effects of tramadol alone. Finally, while considering the experimental design, the use of one subjective outcome, the limited power of analysis and the intra-group variability, the analgesic efficacy assessment has to be considered with prudency. This preliminary study could be claimed as observational, and in consequence is not demonstrating some analgesic efficacy of tramadol in canine OA. Rather, it is highly suggestive that a one-month continuous treatment combining tramadol to NSAID is safe and beneficial for analgesia induction and persistence after treatment disruption.

## Conclusions

This study shows that reduced dosage ketoprofen combined or not with tramadol can be safely administered to dogs with OA for up to 28 days. Standard dosage ketoprofen revealed clinically important adverse affects. No deleterious adverse effects were observed with the combination of ketoprofen and tramadol. The analgesic efficacy of the latter, while questioned when used alone over a short period of treatment [18, 19], was indeed evident when tested in association with ketoprofen over 1 month, and it appears that its combination with NSAID to be attractive for canine OA pain management.

## Methods

The study was approved by the local Institutional Animal Care and Use Committee of the VetAgro-Sup of Lyon (protocol JB/EG 14/23/X). It was performed at the *Centre hospitalier universitaire vétérinaire* (CHUV) – Small Animal Clinic and Medical Biology Laboratory, Lyon, France in collaboration with the University of Pisa, Italy. This study is reported according to the CONSORT (www.consort-statement.org) for reporting a randomized trial and ARRIVE guidelines (https://www.nc3rs.org.uk/arrive-guidelines) for reporting animal experiments.

### Inclusion criteria

Client-owned dogs of any sex or breed, older than 1 year of age, weighing from 10 to 60 kg, and presenting clinical and radiographic evidence of OA were included in a randomized prospective controlled double-blinded clinical trial after obtaining owner’s written consent. Clinical evidence of OA was confirmed by a history of mobility impairment in addition to at least one abnormality found on orthopedic examination including lameness, pain or crepitus during joint palpation and manipulation. Radiographic evidence of OA was defined as presence of at least one of the following radiographic lesions observed in one or more joints: osteophytes / enthesophytes, subchondral bone sclerosis and joint effusion according to previously published criteria [47]. Radiographs had to be performed within 3 months from inclusion in the study. In addition, dogs had to be considered healthy based on physical and laboratory examination (see below). Dogs could not have been administered any NSAID or nutritional supplement or chondroprotector for at least 21 days prior to inclusion in the study, nor any intra-articular injection of any compound in the previous 28 days.

Exclusion criteria included gestation, lameness secondary to pathologies other than OA (e.g., trauma, infection, neurological disease), or any other systemic or metabolic condition. These included any evidence of renal, hepatic, GI or coagulation disorder.

### Treatments

A total of 25 dogs were included. Because of the preliminary nature of this safety study, no sample size calculation was possible. Group A was composed of research healthy Beagle dogs for ethical reasons (as ketoprofen product labeling mentioned no indication for use longer than 5 consecutive days, and the expected occurrence of adverse events required a cautious follow-up of these dogs). Client-owned dogs that fit the aforementioned inclusion criteria were randomized (simple randomization using random number table) to groups B and C. The following treatments were administered: Group A (*n* = 5): ketoprofen (Ketofen™, 10 mg/mL, Merial S.A.S., Lyon, France) at 2 mg/Kg SC on day 0 (D0), followed by ketoprofen (Ketofen™ 5, 10 or 20 mg tablet, Merial S.A.S.) at 1 mg/Kg/D PO for 27 days; Group B (*n* = 10): ketoprofen 0.5 mg/Kg SC on D0 followed by 0.25 mg/Kg/D PO for 27 days; Group C (n = 10): ketoprofen (same dosage as Group B) and tramadol (Contramal S.R.™ 100, 150 or 200 mg tablet, Grünenthal S.p.A., Origgio, Italy) at 5 mg/Kg/D PO for 28 days.

The owners and individuals involved with the dogs’ evaluations were not aware of the treatment being administered. Random allocation sequence was provided by the principal investigator. For the oral administration of ketoprofen and tramadol, capsules of different concentrations were used and/or split so that dogs were receiving, in blinded specific packages, the required dosage according to their body weight over the period of 28-days treatment.

### Evaluation time-points

All dogs were evaluated at days (D)0, D7 and D28. At each time-point, a standard physical exam and pain scoring, using the Standardized Veterinarian Arthritis Pain Scale (SVAPS), [48] were performed, followed by a blood sampling for standard hematology and biochemistry analyses, as well as coagulation profile, GI endoscopy and GFR measurement. At D60, physical exam, SVAPS scoring, standard laboratory analyses and coagulation profile were repeated for assessing possible long-term adverse-effects. At D120, a last SVAPS score was completed. At the end of the study, the dogs either returned in the institutional research colony (Group A, *n* = 5) or stayed at home (Groups B and C, *n* = 20).

### Standard bioanalyses

Standard hematology and serum biochemistry analyses were conducted, under good laboratory practice guidelines, in the Medical Biology Laboratory. These included: CBC using Easycell® (Hycel, Paris, France), manual counting by an experienced technician, and platelet function analyzer (PFA – see below). Serum biochemistry profile was established using the Reflotron® analyzer (Scil, Strasbourg, France). Reference values were established in the Medical Biology Laboratory (see Table 1).

### Coagulation profile

Platelet aggregation time (PAT) was assessed after platelet stimulation using a mixture of collagen and adrenaline (PFA-100®, Dade-Behring, Paris) with a maximum cut-off of 300 s. In dogs, a normal PAT value is considered < 210 s [49], whereas internal validation according to our experimental conditions indicated a normal PAT value as < 213 s. Buccal mucosal bleeding time was performed in dogs in which platelet aggregation time was increased and it was considered normal if it was below 4 min.

### Gastrointestinal endoscopy

Endoscopies were performed while dogs were under general anesthesia. Food, and water was withheld for 12, and 2 h, respectively. Briefly, an appropriately sized catheter was placed under aseptic conditions in the cephalic vein, and premedication with diazepam (Valium™ 5 mg/mL, Roche, Boulogne-Billancourt, France) (0.25 mg/Kg IV) was administered, followed by induction with propofol (Rapinovet™ 10 mg/mL, Bayer S.A.S., Lyon, France) (4 mg/kg IV). Endotracheal intubation was performed with an appropriately sized cuffed endotracheal tube and general anesthesia was maintained with isoflurane (Forene™, AbbVie, Rungis, France) as needed, delivered with 100% oxygen. During the procedure, an IV perfusion of Ringer Lactate (10 mL/Kg/h) was administered. For the endoscopy, a gastroscope of 1.3 m, or a pediatric fibroscope, with a cold light source (Eurolight N°202/98; Optomed, Les Ulis, France) via video camera (Eurocam N°9,960,161; Optomed) were used. The image was then digitally reproduced to a screen (Trinitron Color Video Monitor N°2,011,041; Sony, Tokyo, Japan) and printer (Trinitron Color Video Printer N°10,666; Sony).

For each endoscopy, the integrity of the GI mucosa was evaluated [34, 35]. Specifically, the esophagus, body of the stomach, pyloric antrum, duodenum and cardia (seen in retroflexion) were systematically inspected and individually scored according to the lesions observed (Table 4). The sum of the five anatomical regions’ scores was calculated for each time-point.

**Table 4:** Endoscopic score used to grade the severity of gastrointestinal mucosal lesions in dogs. Adapted from [35]

Score	Lesion
0	None
1	Mucosal congestion
2	1 petechia
3	2–5 petechiae
4	6–20 petechiae
5	> 20 petechiae
6	1 suffusion
7	> 1 suffusions
8	Erosion(s)^a^
9	Non-perforating ulcer^b^
10	Perforating ulcer^b^

Five anatomical locations (esophagus, body of the stomach, pyloric antrum, duodenum and cardia) were individually evaluated and scored, before being summated

^a^Erosion was defined as a discontinuation of the mucosal epithelium

^b^Ulcer was defined as a lesion producing a wide discontinuation of the mucosa and having a crate-like centre

### Glomerular filtration rate

Glomerular filtration rate was determined via iohexol plasma clearance, as previously established in the Medical Biology Laboratory [50]. Briefly, serial blood samples were collected before and up to 3 h after tracer IV injection (Omnipaque™ 300 mg/mL, GE Healthcare S.A.S., Velizy-Villacoublay, France). At D0, blood sampling times (T) were 0, 5, 20, 40, 60, 80, 100, 120, 150 and 180 min, and at D7 and D28, they were reduced to T0, 20, 100 and 180 min, with the help of the modelization. Iohexol plasma concentration was determined using X-ray fluorescence [50]. Clearance was normalized to body surface area (BSA), expressed as mL/min/m^2^ with BSA estimated from BW using the Meeh’s formula where BSA = K x (BW)^2/3^ / 10^4.^ Body surface area is expressed in m^2^, BW in g, and K is a constant of 10.1 in dogs, as normalization to BSA was demonstrated to be optimal, compared to BW and extracellular fluid volume [50].

### Outpatient monitoring of adverse events

Owners of dogs in groups B and C were given a questionnaire to be completed at home during the study period which included the following outwardly detectable clinical signs: salivation, nausea, vomiting, diarrhea, hematochezia, constipation, pica, polyuria and polydipsia, oliguria or anuria, changes in behavior or general status (e.g., anorexia, depression, sedation) and neurological signs (e.g., any abnormal behavior, restlessness, difficulty walking, tremors, convulsions). At any time that these clinical signs were observed, owners were asked to report, date and describe such events.

### Pain scoring

A subjective clinical metrology instrument, SVAPS [48], was completed by a blinded (to treatment) veterinarian (CLA) to establish pain score in five different time-points (Additional file 1 – Standardized Veterinarian Arthritis Pain Scale). This clinical metrology instrument has been developed with clinical OA [51], including a global assessment through a five-point Likert scale, emotional functioning of the owner toward his/her OA-afflicted dog, physical functioning, and response to treatment. Subsequently, an updated version was validated on dogs affected with experimental OA [48]. Briefly, the SVAPS pain scale includes a Global assessment numerical rating scale (from 0 to 4, where ‘0’ means no pain, and ‘4’ extreme pain), and assessment for Lameness while Standing-up, Walking and Trotting, Willingness to hold-up contralateral (to the most affected) limb, Reaction to the Palpation / Mobilization of the most affected limb (joint) and Intensity of these reactions. Each of these nine criteria was scored from 0 to 4, giving a total up to 36. The validation included inter- and intra-observer reliability, internal consistency and concurrent validation in comparison to objective outcomes [48]. At D28, the treatment in Groups B and C was discontinued. Up to D120, the owner (under the supervision of the treating veterinarian) had the possibility to administer rescue analgesia (same treatment for 3 to 5 days) when he/she would observe acute OA pain exacerbations, that we called ‘OA pain crisis’.

### Statistical analysis

Data was analyzed using SPSS software (IBM Corp. released 2011, IBM SPSS Statistics for Windows, Version 20.0, Armonk, NY: IBM Corp.). Data were analyzed with a general linear mixed model for repeated measures, with two-tailed testing and an alpha value set at 0.05. Adjustment for multiple comparisons was applied when necessary. The model included the fixed effects of treatment, time point, and interaction between treatment and time point. The random effects included animal, treatment, and error. The best covariance structure (compound symmetry) was chosen according to Akaike information criterion. Change over time was detected by testing for within-time group variation, and treatment effect was detected by significant change over time for groups A, B and C. If any treatment-related effect was significant, pairwise treatment comparisons were done at each time point.

## Additional file

Additional file 1: Standardized Veterinarian Arthritis Pain Scale (SVAPS). The questionnaire used by a blinded veterinarian to assess osteoarthritic pain in dogs is presenting five sections, respectively Global assessment, Lameness, Willingness to hold up contralateral (to the most affected) limb, Reaction to the palpation/ mobilization of the affected area, and Intensity/ nature of this reaction. (DOCX 20 kb)

## Data Availability

The datasets used and analyzed during the current study are available from the corresponding author on reasonable request.

## Abbreviations

BSA: Body surface area
BW: Body weight
CBC: Complete blood count
COX: Cyclooxygenase
D: Day
e.g.: *exempli gratia*
EP4: Prostaglandin E_2_ receptor 4
GFR: Glomerular filtration rate
GI: Gastrointestinal
IV: Intravenous
NSAID: Non-steroidal anti-inflammatory drug
OA: Osteoarthritis
PAT: Platelet aggregation time
PFA: Platelet function analyzer
PO: per os
SVAPS: Standardized veterinarian arthritis pain scale
T: Time
