# Supplementary material for: Safety and efficacy of reduced dosage ketoprofen with or without tramadol for long-term treatment of osteoarthritis in dogs: a randomized clinical trial
Source: BMC Vet Res. 2019 Jun 25;15:213. doi: 10.1186/s12917-019-1960-3 (PMC6591828; doi:10.1186/s12917-019-1960-3)
Supplement: Supplementary file 1 — Standardized Veterinarian Arthritis Pain Scale (SVAPS). The questionnaire used by a blinded veterinarian to assess osteoarthritic pain in dogs is presenting five sections, respectively Global assessment, Lameness, Willingness to hold up contralateral (to the most affected) limb, Reaction to the palpation/ mobilization of the affected area, and Intensity/ nature of this reaction. (DOCX 20 kb) [file 12917_2019_1960_MOESM1_ESM.docx]

**Additional file 1** – Standardized Veterinarian Arthritis Pain Scale (SVAPS)

| **STANDARDIZED EVALUATION SCALE: Subjective evaluation by the veterinarian** | | | | | | | | | | | | | | | | | | |
| --- | --- | --- | --- | --- | --- | --- | --- | --- | --- | --- | --- | --- | --- | --- | --- | --- | --- | --- |
|  |  | |  | |  | | | |  | |  | | | | |  |  |  |
| **Global assessment** | | | |  | | | | |  | |  | | | | |  |  |  |
| ***Today overall veterinarian assessment*** | | | |  | | | | | | | | | | | |  |  |  |
|  | Pain | Absent | |  | |  | | | | | |  | | | | Extreme |  |  |
|  | Score | 0 | | 1 | | 2 | | | | | | 3 | | | | 4 |  |  |
|  |  |  | |  | |  | | | | | |  | | | |  |  |  |
| **Lameness** | | | | | |  | | | | | |  | | | |  |  |  |
|  |  | |  | | | |  |  | | | |  | | | |  |  |  |
|  | | | | | | |  |  | | **Standing-up** | | | | | | **Walking** | **Trotting** |  |
| Full, constant weight bearing | | | | | | |  |  | | 0 | | | | | | 0 | 0 |  |
| Sometimes full, sometimes partial weight bearing | | | | | |  | | | | 1 | | | | | | 1 | 1 |  |
| Partial, constant weight bearing | | | | | | | | | | 2 | | | | | | 2 | 2 |  |
| Sometimes partial, sometimes no weight bearing | | | | | | | | | | 3 | | | | | | 3 | 3 |  |
| No weight bearing at all in the right hind limb | | | | | | | | | | 4 | | | | | | 4 | 4 |  |
|  |  | |  | | | |  | |  | | |  | | | |  |  |  |
| **Willingness to hold up contralateral (to the most affected) limb^1^** | | | | | | | | |  | | | |  |  | | |  |  |
|  | | | | | | |  | |  | |  | | |  | | |  |  |
| Readily accepts contralateral limb being held up and bears full weight on affected limb | | | | | | | | |  | | 0 | | |  | | |  |  |
| Offers some resistance to elevation of contralateral limb, but bears full weight on affected limb for more than 1 min after contralateral limb is elevated | | | | | | | | | | | 1 | | |  | | |  |  |
| Offers moderate resistance to elevation of contralateral limb and replaces it after 30 sec. | | | | | | | | | | | 2 | | |  | | |  |  |
| Offers strong resistance to elevation of contralateral limb, and replaces it after 10 sec. | | | | | | | | | | | 3 | | |  | | |  |  |
| Refuses to raise contralateral limb | | | | | | |  | |  | | 4 | | |  | | |  |  |
| ^1^*The contralateral limb must be elevated at the level of the vertebra column for 1 min max, once the more affected limb has been detected from the first two assessments (i.e. Global assessment, and Lameness while Standing-ip, Walking and Trotting), if possible.* | | | | | | | | | | | | | | | | | | |
|  |  | |  | | | |  | |  | |  | | | |  | |  |  |

| **Reaction to the palpation^2^/ mobilization^3^ of the affected area** | | | | | | | | |  | | |  | |
| --- | --- | --- | --- | --- | --- | --- | --- | --- | --- | --- | --- | --- | --- |
| No visible or audible reaction | | | |  |  | **Palpation**^2^ | | | | **Mobilization^3^** | |  | |
|  | After 4 manipulations | |  |  |  | 0 | | | | 0 | |  | |
| Visible or audible reaction(s) | | |  |  |  |  | | | |  | |  | |
|  | At the 4^th^ manipulation | |  |  |  | 1 | | | | 1 | |  | |
|  | At the 3^rd^ manipulation | |  |  |  | 2 | | | | 2 | |  | |
|  | At the 2^nd^ manipulation | |  |  |  | 3 | | | | 3 | |  | |
|  | At the 1^st^ manipulation or non-evaluable^4^ | |  |  |  | 4 | | | | 4 | |  | |
|  |  | |  |  |  |  | | | |  | |  | |
| *^2^The operator begins with palpating the contralateral (to the most affected) limb from the proximal part to the extremity (carpum/ tarsum); after that, he/ she is doing the same to the most affected limb with 4 (if possible) palpations of the most affected joint;*  *^3^ The operator manipulates the affected joint by closing it in flexion for 3 seconds before putting it back to normal and repeating the flexion for 4 (if possible) flexions of the joint. He/ she is finishing with a small extension of the joint;* | | | | | | | | | | | |  | |
| *^4^Dog will not allow the examiner to palpate joint.* | | | | |  | |  |  | | |  | |  |
|  |  | |  |  |  |  | |  | | |  | |  |
| **Intensity/ nature of this reaction** | | | | | |  | |  | | |  | |  |
|  |  | |  |  |  | **Palpation^2^** | | **Mobilization**^3^ | | |  | |  |
| No response | |  |  |  |  | 0 | | 0 | | |  | |  |
| Mild response: | |  |  |  |  | 1 | | 1 | | |  | |  |
| *turns head in recognition, tries a protective (guarding) posture* | | | | | |  | |  | | |  | |  |
| Moderate response: | | |  |  |  | 2 | | 2 | | |  | |  |
| *turns head, vocalizes (complaining), pulls limb away* | | | | |  |  | |  | | |  | |  |
| Severe response: | | |  |  |  | 3 | | 3 | | |  | |  |
| *vocalizes, bites, licks or scratches at the joint, becomes aggressive* | | | | | |  | |  | | |  | |  |
| Extreme response: | | |  |  |  | 4 | | 4 | | |  | |  |
| *non-evaluable^4^* |  | |  |  |  |  | |  | | |  | |  |
|  |  | |  |  |  |  | |  | | |  | |  |
| **TOTAL (0 to 36) =** | | |  |  |  |  | |  | | |  | |  |
